# Differential tDCS and tACS Effects on Working Memory-Related Neural Activity and Resting-State Connectivity

**DOI:** 10.3389/fnins.2019.01440

**Published:** 2020-01-17

**Authors:** Kilian Abellaneda-Pérez, Lídia Vaqué-Alcázar, Ruben Perellón-Alfonso, Núria Bargalló, Min-Fang Kuo, Alvaro Pascual-Leone, Michael A. Nitsche, David Bartrés-Faz

**Affiliations:** ^1^Department of Medicine, Faculty of Medicine and Health Sciences, Institute of Neurosciences, University of Barcelona, Barcelona, Spain; ^2^Institute of Biomedical Research August Pi i Sunyer, Barcelona, Spain; ^3^Hospital Clínic de Barcelona, Magnetic Resonance Image Core Facility, Institute of Biomedical Research August Pi i Sunyer, Barcelona, Spain; ^4^Hospital Clínic de Barcelona, Neuroradiology Section, Radiology Service, Centre de Diagnòstic per la Imatge, Barcelona, Spain; ^5^Leibniz Research Centre for Working Environment and Human Factors, Dortmund, Germany; ^6^Hinda and Arthur Marcus Institute for Aging Research, Hebrew SeniorLife, Boston, MA, United States; ^7^Department of Neurology, Harvard Medical School, Boston, MA, United States; ^8^Guttmann Brain Health Institute, Institut Universitari de Neurorehabilitació Guttmann, Autonomous University of Barcelona, Bellaterra, Spain; ^9^Department of Neurology, University Medical Hospital Bergmannsheil, Bochum, Germany

**Keywords:** transcranial direct current stimulation (tDCS), transcranial alternating current stimulation (tACS), resting-state functional magnetic resonance imaging (rs-fMRI), task-based functional magnetic resonance imaging (tb-fMRI), working memory (WM)

## Abstract

Transcranial direct and alternating current stimulation (tDCS and tACS, respectively) entail capability to modulate human brain dynamics and cognition. However, the comparability of these approaches at the level of large-scale functional networks has not been thoroughly investigated. In this study, 44 subjects were randomly assigned to receive sham (*N* = 15), tDCS (*N* = 15), or tACS (*N* = 14). The first electrode (anode in tDCS) was positioned over the left dorsolateral prefrontal cortex, the target area, and the second electrode (cathode in tDCS) was placed over the right supraorbital region. tDCS was delivered with a constant current of 2 mA. tACS was fixed to 2 mA peak-to-peak with 6 Hz frequency. Stimulation was applied concurrently with functional magnetic resonance imaging (fMRI) acquisitions, both at rest and during the performance of a verbal working memory (WM) task. After stimulation, subjects repeated the fMRI WM task. Our results indicated that at rest, tDCS increased functional connectivity particularly within the default-mode network (DMN), while tACS decreased it. When comparing both fMRI WM tasks, it was observed that tDCS displayed decreased brain activity post-stimulation as compared to online. Conversely, tACS effects were driven by neural increases online as compared to post-stimulation. Interestingly, both effects primarily occurred within DMN-related areas. Regarding the differences in each fMRI WM task, during the online fMRI WM task, tACS engaged distributed neural resources which did not overlap with the WM-dependent activity pattern, but with some posterior DMN regions. In contrast, during the post-stimulation fMRI WM task, tDCS strengthened prefrontal DMN deactivations, being these activity reductions associated with faster responses. Furthermore, it was observed that tDCS neural responses presented certain consistency across distinct fMRI modalities, while tACS did not. In sum, tDCS and tACS modulate fMRI-derived network dynamics differently. However, both effects seem to focus on DMN regions and the WM network-DMN shift, which are highly affected in aging and disease. Thus, albeit exploratory and needing further replication with larger samples, our results might provide a refined understanding of how the DMN functioning can be externally modulated through commonly used non-invasive brain stimulation techniques, which may be of eventual clinical relevance.

## Introduction

Working memory (WM) provides temporary storage and manipulation of information required for a variety of complex cognitive tasks (Baddeley, 1992, 2010). WM capacity plays a central role in daily life activities and is predictive for a wide-range of higher-level cognitive measures (Johnson et al., 2013; Unsworth et al., 2014). Impairments in WM entail functionally disabling symptoms in advanced age (Park et al., 2002; Park and Reuter-Lorenz, 2009; Anderson and Craik, 2017) and in several neuropsychiatric conditions (Lee and Park, 2005; Nakao et al., 2009).

The WM network (WMN) includes a fronto-parietal loop (Owen et al., 2005), where the dorsolateral prefrontal cortex (dlPFC) is of particular relevance (Curtis and D’Esposito, 2003; Barbey et al., 2013). This fronto-parietal circuit shows a negative correlation with the default-mode network (DMN; Fox et al., 2005; Buckner et al., 2008; Raichle, 2015). The DMN has been shown to be consistently activated during rest, while its nodes are inhibited during externally oriented tasks (Fox et al., 2005; Pfefferbaum et al., 2011). Thus, the brain may shift between two modes of information processing, one that puts the attentional focus on external stimuli and another one that relates to internally directed processing (Buckner et al., 2008). At the electrophysiological level, it has been shown that WM processes are mediated by synchronous firing of neural populations at distinct frequencies as well as via cross-frequency coupling (Sarnthein et al., 1998; Howard et al., 2003; Sauseng et al., 2005; Jensen and Colgin, 2007; Lisman and Jensen, 2013; Roux and Uhlhaas, 2014). A large body of literature indicates that the coupling of theta and gamma oscillations mediates communication within and between brain networks in general and during WM tasks in particular, possibly accounting for WM processing and capacity demands (for a review see Lisman and Jensen, 2013; Hanslmayr et al., 2019).

Despite the fact that neuroimaging and neurophysiological investigations have been providing relevant data on the anatomo-functional correlates of WMN and its anticorrelated systems (Nee et al., 2013; Roux and Uhlhaas, 2014; Eriksson et al., 2015; Raichle, 2015), the cognitive benefits derived from interventional approaches aimed to improve WM functioning have been limited. Notwithstanding, new methodologies, such as transcranial electrical stimulation (tES), have recently shown potential to enhance WM performance by targeting its critical network hubs, such as the dlPFC (Fregni et al., 2005; Ohn et al., 2008; Zaehle et al., 2011; Brunoni and Vanderhasselt, 2014; Meiron and Lavidor, 2014; Hoy et al., 2015; Alekseichuk et al., 2016; Dedoncker et al., 2016). Nevertheless, and despite this promising developments, data is still inconclusive, particularly in healthy populations (Tremblay et al., 2014; Hsu et al., 2015; Nilsson et al., 2015; Mancuso et al., 2016; Hill et al., 2017; Medina and Cason, 2017; Imburgio and Orr, 2018).

Among tES techniques, transcranial direct current stimulation (tDCS) and transcranial alternating current stimulation (tACS) are the most commonly used (Polanía et al., 2018). tDCS delivers weak tonic currents to the scalp. During tDCS, neural membrane potentials are depolarized under the anode, leading to an increase in cortical excitability, while neural membrane hyperpolarization develops under the cathode, thereby diminishing cortical excitability (Purpura and McMurtry, 1965; Nitsche and Paulus, 2000; Nitsche et al., 2008). On the other hand, tACS applies a sinusoidal current to the scalp at specific frequencies, exerting an exogenous modulation of ongoing brain oscillations (Zaghi et al., 2010a; Ali et al., 2013; Antal and Paulus, 2013; Herrmann et al., 2013; Reato et al., 2013; Antal and Herrmann, 2016; Moisa et al., 2016). Beyond their immediate impact, both techniques display after-effects that can outlast the period of stimulation, probably due to their capability to induce neuroplasticity-like processes (Nitsche and Paulus, 2001; Liebetanz et al., 2002; Nitsche et al., 2003; Monte-Silva et al., 2013; Vossen et al., 2015; Kasten et al., 2016; Wischnewski et al., 2019).

In this context, only two studies have explored the differential impact of tDCS and tACS on WM performance (Hoy et al., 2015; Röhner et al., 2018), indicating a more relevant effect of tACS as compared to tDCS. However, the physiological underpinnings of those protocols over large-scale neural systems supporting the WM function remain understudied. Further, to our knowledge, a direct comparison of the effects of these different stimulation protocols at the functional magnetic resonance imaging (fMRI)-derived network level has not been so far investigated. Since aging and various neuropsychiatric disorders show alterations in WM circuits and may benefit from their modulation, a better insight on tES impact on the brain’s WMN and its linked neural systems, such as the DMN, would likely have clinical translational relevance.

## Materials and Methods

### Participants

Forty-four healthy young subjects [age mean ± standard deviation (*SD*), 25.25 ± 4.22 years; age range, 19–37 years; 20 females; years of education mean ± *SD*, 21.11 ± 3.40 years; 36 right-handed] naive to tES were recruited from the general population and provided informed consent to participate in this study, in accordance with the Declaration of Helsinki (1964, last revision 2013). All study procedures were approved by the Institutional Review Board (IRB 00003099). None of the participants reported a diagnosis of a neurological or psychiatric disorder. For all participants, MRI images were examined by a senior neuroradiologist for any clinically significant pathology (none found).

### Experimental Design

**FIGURE 1:**
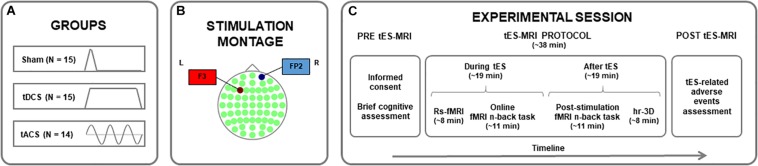
Study protocol. **(A)** Assignment of participants to one of the experimental groups. **(B)** Stimulation montage for all groups, with the first electrode (anode for tDCS) centered over the F3 (in red) and the second electrode (cathode for tDCS) placed over the FP2 (in blue) in a 10–10 system map. **(C)** Timeline of the procedures accomplished before, during and after the tES-MRI protocol. tDCS, transcranial direct current stimulation; tACS, transcranial alternating current stimulation; tES, transcranial electrical stimulation; MRI, magnetic resonance imaging; rs-fMRI, resting-state functional MRI; hr-3D, high-resolution three-dimensional.

The present study was conducted in a randomized between-subjects placebo-controlled design. Online effects of prefrontal tDCS and tACS on resting-state fMRI (rs-fMRI) were assessed. Furthermore, the online and post-stimulation impact of these intervention protocols on WM-related neural activity and performance was explored, using a similar experimental setting as described elsewhere (i.e., Meiron and Lavidor, 2014; Brauer et al., 2018). Participants were randomly assigned to receive sham stimulation (*N* = 15), anodal tDCS (*N* = 15) or theta tACS (*N* = 14; Figure 1A) over the left dlPFC (l-dlPFC; Figure 1B; see also see section “Transcranial Electrical Stimulation (tES) Parameters”). A simple randomization procedure was used (Altman and Bland, 1999; Kang et al., 2008).

First, a brief cognitive assessment was conducted to obtain an estimation of the intelligence quotient (IQ) of the participants, using the vocabulary subtest of the Wechsler Adult Intelligence Scale-IV (WAIS-IV). Subsequently and within the same experimental day, tES was applied inside an MRI scanner. Here, an rs-fMRI sequence (∼8 min) was acquired before subjects underwent two sequential task-based fMRI (tb-fMRI) acquisitions while performing two identical verbal n-back tasks (∼11 min each one; Sala-Llonch et al., 2012). The rs-fMRI and the first tb-fMRI datasets were acquired during stimulation (i.e., online fMRI n-back task) and the second tb-fMRI sequence after stimulation delivery was turned off (i.e., post-stimulation fMRI n-back task). A high-resolution three-dimensional (hr-3D) structural image (∼8 min) was acquired at the end of the MRI session for co-registration purposes. A questionnaire of tES-related adverse events was administered at the end of the experimental session [adapted from Brunoni et al., 2011; Figure 1C; see also Supplementary Material (SM)].

### Transcranial Electrical Stimulation (tES) Parameters

Stimulation was delivered using a battery-driven MRI-compatible DC-Stimulator Plus (neuroConn GmbH, Ilmenau, Germany) and was transferred by two MRI-compatible conductive rubber electrodes (7 cm × 5 cm) positioned in a room adjacent to the MRI scanner. The same montage was used in all groups, as applied in similar recent comparative studies (Lang et al., 2019). According to the international 10–10 system of measurement, the first electrode (anode in tDCS) was positioned over the F3 (l-dlPFC) and the second electrode (cathode in tDCS) was placed over the FP2 (right supraorbital area). This is one of the standard montages frequently employed to stimulate the l-dlPFC (Fregni et al., 2005; Nitsche et al., 2008; Ohn et al., 2008), the target area. In all groups, the current was initially increased and finally decreased in a ramp-like fashion of 15 s. In the sham condition, the current delivery was terminated after 30 s of stimulation with no further blinding processes. In the real stimulation groups, the current was supplied during 20 min, which covered the rs-fMRI acquisition and the first tb-fMRI sequence. tDCS was delivered with a constant current of 2 mA. tACS was fixed to 2 mA peak-to-peak in a 6 Hz frequency. We selected 6 Hz as this frequency has been widely used in recent tACS WM investigations (i.e., Polanía et al., 2012; Alekseichuk et al., 2016; Violante et al., 2017; Brauer et al., 2018; Röhner et al., 2018; Lang et al., 2019). All stimulation parameters adhered to safety criteria guidelines (Zaghi et al., 2010b; Fertonani et al., 2015; Bikson et al., 2016; Woods et al., 2016; Matsumoto and Ugawa, 2017; see SM for more details).

### N-Back Task

Subjects performed a verbal n-back task, a commonly used paradigm to investigate WM in fMRI (Owen et al., 2005). The n-back task had different levels of memory load (from 1 to 3 letters to be retained) and a basic level of target stimulus identification that were randomly presented during the two consecutive tasks achieved inside the MRI scan (Sala-Llonch et al., 2012; see SM for more information).

### MRI Acquisition

All participants were scanned with a Siemens Magnetom Trio Tim Syngo 3 Tesla system using an 8-channel head coil at the Magnetic Resonance Image Core Facility (IDIBAPS) at the Hospital Clínic de Barcelona, Barcelona, Spain. The imaging sequences were acquired with the following parameters. First, a rs-fMRI dataset (T2^∗^-weighted GE-EPI sequence; interleaved acquisition; repetition time [TR] = 2,700 ms; echo time [TE] = 30 ms; 40 slices per volume; slice thickness = 3.0 mm; interslice gap = 15%; voxel size = 3.0 mm × 3.0 mm × 3.0 mm; field of view [FOV] = 216 mm; 178 volumes) was acquired. Later, two identical fMRI n-back task datasets (T2^∗^weighted EPI scans; interleaved acquisition; TR = 2,000 ms; TE = 28 ms; 34 slices per volume; slice thickness = 3.5 mm; interslice gap = 15%; voxel size = 3.5 mm × 3.5 mm × 3.5 mm; FOV = 238 mm; 336 volumes) were acquired, one during stimulation and another one after stimulation cessation. Lastly, a hr-3D structural dataset (T1-weighted magnetization-prepared rapid gradient-echo [T1-weighted MPRAGE]; sagittal plane acquisition; TR = 2,300 ms; TE = 2.98 ms; inversion time [IT] = 900 ms; slice thickness = 1.0 mm; voxel size = 1.0 mm × 1.0 mm × 1.0 mm; FOV = 256 mm; 240 slices) was acquired.

### Image Analyses

The FMRIB Software Library (FSL; version 6.00^1^) and the Analysis of Functional NeuroImages (AFNI^2^) were used for preprocessing and analyzing neuroimaging data. Rs- and tb-fMRI preprocessing pipelines and head movement considerations are described in SM.

#### Functional Connectivity Analyses

**FIGURE 2:**
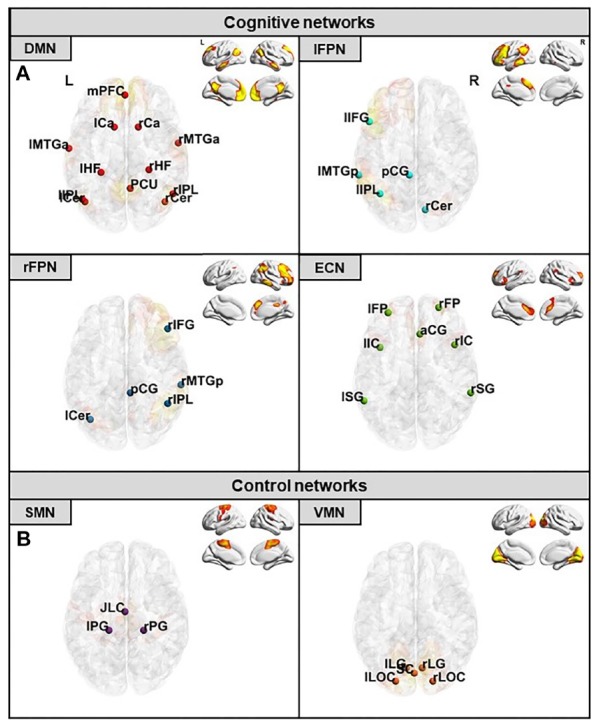
Selected networks and their respective ROIs location. **(A)** Cognitive networks with their corresponding ROIs in red for the DMN, in light blue for the lFPN, in dark blue for the rFPN, and in green for the ECN. **(B)** Control networks with their corresponding ROIs in purple for the SMN and in orange for the VMN. DMN, default-mode network; lFPN, left fronto-parietal network; rFPN, right fronto-parietal network; ECN, executive-control network; SMN, sensorimotor network; VMN, visual-medial network. For ROI abbreviations see Table 1.

Resting-state functional connectivity (rs-FC) analyses were performed using a seed-to-seed approach, following previous procedures in our group (i.e., Abellaneda-Pérez et al., 2019). Firstly, the concatenated fMRI dataset containing all rs-fMRI acquisitions from the entire sample was decomposed through independent component analysis (ICA) into 15 components using the Multivariate Exploratory Linear Optimized Decomposition into Independent Components (MELODIC; version 3.15) algorithm, part of the FSL (Smith et al., 2004; Beckmann et al., 2005; Jenkinson et al., 2012). The components related to the archetypical resting-state network, namely the DMN (Fox et al., 2005; Buckner et al., 2008; Raichle, 2015), along with the WM-related systems, the left and right fronto-parietal networks (lFPN and rFPN, respectively), were selected in a similar manner as described in previous reports from our group (i.e., Sala-Llonch et al., 2012). Furthermore, the executive-control network (ECN) was additionally considered due to its prefrontal nodes and its known relevance in cognitive functions (Smith et al., 2009). Moreover, two components that do not include the l-dlPFC and are not related to cognitive processing were used as control networks, namely the sensorimotor and visual-medial networks (SMN and VMN, respectively), as has been done in previous work of our group (i.e., Peña-Gómez et al., 2012). All components were identified using spatial correlations against previously defined maps (Smith et al., 2009). Secondly, the main regions of interest (ROIs) were selected based on the peak voxels of each network (Figure 2 and Table 1). Next, spherical seeds with a 6-mm radius were placed over the identified regions, and ROI-specific time-series from the preprocessed and regressed data were extracted. Finally, to obtain an rs-FC measure for each seed-to-seed coupling in each subject, obtained ROI time-series were correlated with each other within every network using Pearson’s correlation coefficients.

**TABLE 1:** Selected networks and their respective ROIs with associated coordinates in the Montreal Neurological Institute (MNI) system.

**RSNs**	**MNI coordinates**			**ROI**	**ROI abbreviated**
	***X***	***Y***	***Z***		
DMN	0	51	−15	Medial prefrontal cortex	mPFC
	6	−54	21	Precuneus cortex	PCU
	−48	−66	30	Left inferior parietal lobule	lIPL
	54	−60	27	Right inferior parietal lobule	rIPL
	−63	−9	−21	Left middle temporal gyrus (anterior division)	lMTGa
	60	−3	−24	Right middle temporal gyrus (anterior division)	rMTGa
	−12	15	3	Left caudate	lCa
	15	15	6	Right caudate	rCa
	−27	−36	−18	Left hippocampal formation	lHF
	27	−33	−15	Right hippocampal formation	rHF
	−45	−69	−42	Left cerebellum	lCer
	45	−69	−42	Right cerebellum	rCer^∗^
lFPN	−51	21	21	Left inferior frontal gyrus	lIFG
	−39	−60	42	Left inferior parietal lobule	lIPL
	−63	−39	−9	Left middle temporal gyrus (posterior division)	lMTGp
	−6	−39	33	Cingulate gyrus (posterior division)	pCG
	12	−78	−30	Right cerebellum	rCer
rFPN	48	33	27	Right inferior frontal gyrus	rIFG
	48	−51	48	Right inferior parietal lobule	rIPL
	63	−30	−15	Right middle temporal gyrus (posterior division)	rMTGp
	6	−39	36	Cingulate gyrus (posterior division)	pCG
	−39	−69	−48	Left cerebellum	lCer
ECN	6	27	24	Cingulate gyrus (anterior division)	aCG
	−30	51	18	Left frontal pole	lFP
	27	57	18	Right frontal pole	rFP
	−39	12	−6	Left insular cortex	lIC
	45	15	−6	Right insular cortex	rIC
	−57	−48	30	Left supramarginal gyrus	lSG
	63	−39	33	Right supramarginal gyrus	rSG
SMN	0	−9	51	Juxtapositional lobule cortex	JLC
	−18	−30	63	Left precentral gyrus	lPG
	21	−30	60	Right precentral gyrus	rPG
VMN	0	−78	18	Supracalcarine cortex	SC
	−21	−87	27	Left lateral occipital cortex	lLOC^∗^
	21	−87	27	Right lateral occipital cortex	rLOC
	−9	−72	0	Left lingual gyrus	lLG
	9	−72	0	Right lingual gyrus	rLG^∗^

*The asterisk (^∗^) represents those ROIs that were not identified based on the functional peak voxels but selected for anatomo-functional reasons regarding the coordinates of the homologous region in the other hemisphere, being the two ROIs inside the network in the most anatomically plausible position. RSNs, resting-state networks; DMN, default-mode network; lFPN, left fronto-parietal network; rFPN, right fronto-parietal network; ECN, executive-control network; SMN, sensorimotor network; VMN, visual-medial network; MNI, Montreal Neurological Institute; ROI, region of interest.*

#### N-Back fMRI Data

Tb-fMRI data were analyzed with the FEAT-FSL software (Smith et al., 2004). At the first-level analysis, data were fit into a general linear model (GLM) containing the task time-series with a gamma convolution of the hemodynamic response function (Woolrich et al., 2001). In this GLM, four regressors and their first temporal derivatives were modeled: 0-back, 1-back, 2-back, and 3-back. We defined three contrasts of interest combining the distinct loads, as the difference of brain activity between 1-back, 2-back, and 3-back and the lowest load (0-back), namely: (1) lowest WM load: 1 > 0-back; (2) intermediate WM load: 2 > 0-back; and (3) highest WM load: 3 > 0-back. The results of the first-level analysis were further fit into higher-level or group-level statistics, performed using the FMRIB’s Local Analysis of Mixed Effects (FLAME; Woolrich et al., 2004). We conducted GLM matrices modeling the different tES time-points (online and post-stimulation) and experimental groups (sham, tDCS, and tACS). Using this second-level GLM and the appropriate contrasts, we evaluated: (1) the group-mean activity maps of the three selected contrasts of interest in the first-level analysis (1 > 0-back, 2 > 0-back, and 3 > 0-back), in order to explore the WM-related neural patterns for each one in every tES time-point; (2) the interactions between tES time-point as a within-subject factor and group as a between-subject factor; (3) the patterns of time-related change within each group, as pairwise paired-samples *t*-tests; and (4) the group differences in each tES time-point separately, exploring neural changes in the following contrasts: tDCS vs. sham, tACS vs. sham and tDCS vs. tACS, as pairwise independent-samples *t*-tests. These analyses were performed voxel-wise and the statistical significance of the resulting maps was set at *p* ≤ 0.05 and *z* ≥ 2.3 (cluster-wise corrected).

### Statistical Analyses

Non-imaging data analyses were performed using IBM SPSS (IBM, Corp., Released 2016. IBM SPSS Statistics for Windows, Version 24.0. Armonk, NY, United States: IBM, Corp.) and MATLAB (Version R2019a, The MathWorks, Inc., Natick, MA, United States). To evaluate differences between groups (sham, tDCS, and tACS) in seed-to-seed rs-FC connections, a one-factorial analysis of variance (ANOVA) was conducted, and all *post hoc* pairwise comparisons were subjected to Bonferroni correction. This statistical procedure was also used when considering tES-related adverse events. To evaluate differences in cognitive performance, a univariate ANOVA was conducted with tES time-point (online and post-stimulation) as a within-subject factor and group (sham, tDCS, and tACS) as a between-subject factor. Following this ANOVA, if there were significant interactions, two pairwise analyses were conducted. First, paired-samples *t*-tests were conducted to assess differences in performance over time in each group. Second, independent-samples *t*-tests were conducted to compare performance between groups for each tES time-point separately. Further, to obtain summary statistics, we extracted the mean values of the blood oxygen level dependent (BOLD) signal from the fMRI clusters derived from the significant neuroimaging results. These data were used to plot the fMRI findings and to corroborate the obtained results. Moreover, these data were used to associate the fMRI BOLD signal with cognitive performance estimates and rs-FC data using Pearson’s correlations. All non-imaging statistical analyses were two-tailed and α was set at 0.05 (see SM for further details).

## Results

### Demographics and N-Back Task Performance

No differences in age, gender, years of education, laterality and premorbid intelligence were found between groups (all *p* values > 0.05; see Table 2). Behaviorally, we did not observe any significant difference in n-back task performance between tES time-points and experimental groups (Supplementary Figure S1).

**TABLE 2:** Demographics and neuropsychological data.

	**Total**	**Sham**	**tDCS**	**tACS**
	**(*N* = 44)**	**(*N* = 15)**	**(*N* = 15)**	**(*N* = 14)**
Age	25.25 ± 4.22	25.40 ± 3.16	24.33 ± 4.12	26.07 ± 5.31
Gender (female/male)	20/24	8/7	7/8	5/9
Years of education	21.11 ± 3.40	21.00 ± 1.73	20.87 ± 4.50	21.50 ± 3.61
Laterality (right-/left-handed)	36/8	13/2	11/4	12/2
Vocabulary WAIS-IV	44.16 ± 4.40	43.60 ± 2.80	42.93 ± 5.86	46.07 ± 3.54

*Data are presented as mean ± *SD* for the whole sample and considering the three experimental groups. WAIS-IV, Wechsler Adult Intelligence Scale-IV; tDCS, transcranial direct current stimulation; tACS, transcranial alternating current stimulation.*

### Effects of tDCS and tACS on rs-FC

**FIGURE 3:**
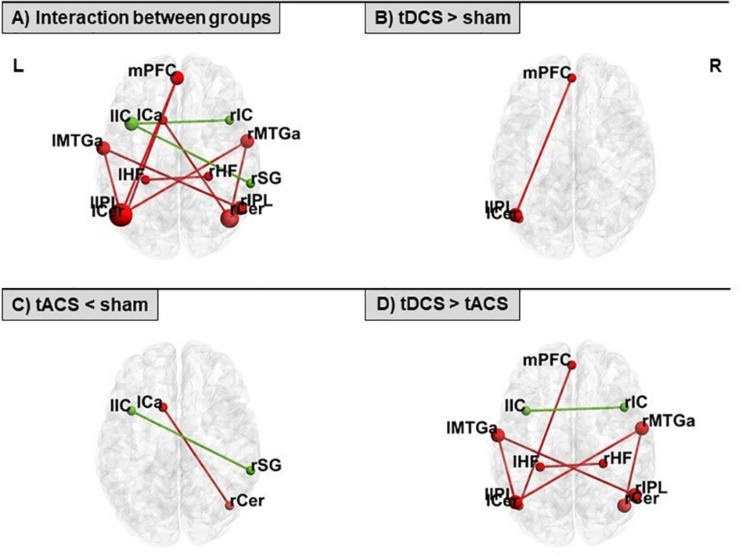
Seed-to-seed statistically significant results within the DMN (in red) and the ECN (in green) for: **(A)** interaction between groups, **(B)** tDCS > sham, **(C)** tACS < sham, and **(D)** tDCS > tACS. tDCS, transcranial direct current stimulation; tACS, transcranial alternating current stimulation. For ROI abbreviations see Table 1.

Rs-FC was differentially modulated with respect to the specific tES protocol. An interaction between groups revealed different connectivity of distinct couplings within the DMN and the ECN. Comparing the real tES groups with sham we observed higher rs-FC in the tDCS group and lower connectivity in the tACS group. Contrasting across the two real tES groups, we observed higher rs-FC in the tDCS group as compared to the tACS group (Figure 3 and Table 3). No differences between groups were found for left and right FPNs. Control networks also remained unmodified.

**TABLE 3:** Seed-to-seed connections with statistically significant interactions and subsequent significant pairwise *post hoc* analyses within the DMN and the ECN.

**RSNs**	**Seed-to-seed coupling**	**Interaction between groups**	**tDCS > sham**	**tACS < sham**	**tDCS > tACS**
DMN	mPFC-lIPL	*F* = 4.510, *p* = 0.017	*p* = 0.021	−	−
	mPFC-lCer	*F* = 5.089, *p* = 0.011	−	−	*p* = 0.009
	lIPL-lMTGa	*H* = 10.960, *p* = 0.004	−	−	*p* = 0.003
	lIPL-rMTGa	*F* = 5.569, *p* = 0.007	−	−	*p* = 0.008
	lIPL-lCer	*F* = 3.525, *p* = 0.039	*p* = 0.037	−	−
	rIPL-lMTGa	*H* = 6.904, *p* = 0.032	−	−	*p* = 0.033
	rIPL-rCer	*F* = 3.352, *p* = 0.045	−	−	*p* = 0.043
	rMTGa-rCer	*F* = 4.495, *p* = 0.017	−	−	*p* = 0.020
	lCa-rCer	*H* = 13.020, *p* = 0.001	−	*p* = 0.001	−
	lHF-rHF	*F* = 3.371, *p* = 0.044	−	−	*p* = 0.046
ECN	lIC-rIC	*H* = 8.502, *p* = 0.014	−	−	*p* = 0.013
	lIC-rSG	*H* = 7.875, *p* = 0.020	−	*p* = 0.016	–

*RSNs, resting-state networks; DMN, default-mode network; ECN, executive-control network; tDCS, transcranial direct current stimulation; tACS, transcranial alternating current stimulation. For ROI abbreviations see Table 1.*

### Effects of tDCS and tACS on WM-Related Neural Activity

An interaction between tES time-points and experimental groups was found in the precuneus (PCU) cortex and the posterior cingulate gyrus in the 2 > 0-back contrast (Supplementary Figure S2A). Further, another interaction was observed in the lateral occipital and the angular gyrus in the 3 > 0-back contrast (Supplementary Figure S2B). The group-mean activity maps obtained from each contrast (1 > 0-back, 2 > 0-back, and 3 > 0-back) in the sham group (considered as the ‘reference’ WM brain patterns) are additionally displayed in SM (Supplementary Figure S3). These maps show the characteristic fronto-parietal WM patterns and the expected task-deactivations, mainly placed within DMN-related areas, like the medial prefrontal cortex (mPFC) and the PCU.

#### Differential Effects Across tES Time-Points

**FIGURE 4:**
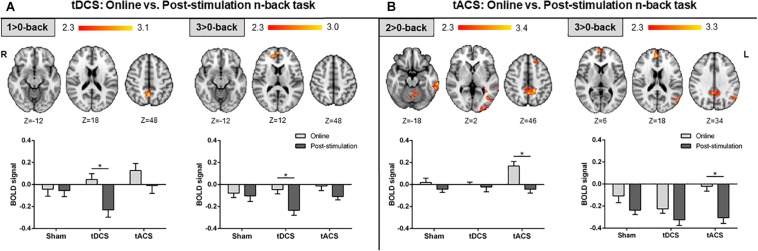
Comparison between the online and post-stimulation fMRI n-back tasks in each group. **(A)** Online vs. post-stimulation results in the tDCS group. **(B)** Online vs. post-stimulation results in the tACS group. Top: statistically significant fMRI activity maps on the standard MNI for each contrast of interest. Results are shown in red-yellow for higher activations (or lower deactivations) online as compared to post-stimulation. Down: plots of mean BOLD signal values at the fMRI clusters considering sham, tDCS and tACS where significant differences between both fMRI n-back tasks were found. Data are presented as mean with standard error of the mean (*SEM*). tDCS, transcranial direct current stimulation; tACS, transcranial alternating current stimulation; BOLD, blood oxygen level dependent.

The active stimulation groups showed differential changes in brain activity between both tES time-points. No time-related fMRI variances were found in sham participants. Specifically, neural modulations in the tDCS group were driven by clear decreases in brain activity post-stimulation as compared to online. These modulations were noticeable in view of the slight neural differences across time in the sham group within the observed significant areas. Anatomically, these changes were found in posterior midline structures during the lowest WM load and within medial frontal areas during the highest WM load (Figure 4A). Conversely, tACS effects appeared to be directed by manifest neural increases and robust lower deactivations online as compared to post-stimulation, in view of the minor brain activity changes in the sham group across time in the detected significant regions. At the anatomical level, increased neural activity was mainly evident during the intermediate WM load in the PCU, frontal and temporal regions, and the occipital lobe. Reduced deactivations were detected in the highest WM load in the medial frontal, posterior midline structures, and the left inferior parietal lobe (lIPL; Figure 4B). It is worth noticing that numerous of the detected fMRI clusters correspond to the main nodes of the DMN (i.e., those areas entailing typical task-deactivation processes).

#### Differential Online Effects

**FIGURE 5:**
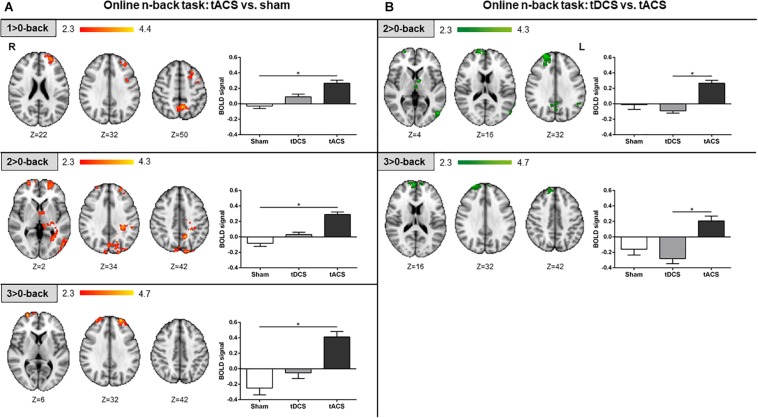
Online fMRI n-back task results. **(A)** tACS vs. sham results are shown in red-yellow. **(B)** tDCS vs. tACS results are shown in green. Left: statistically significant fMRI activity maps on the standard MNI for each contrast of interest and group comparison. Right: plots of mean BOLD signal values at the fMRI clusters considering sham, tDCS and tACS where significant differences between groups were found. Data are presented as mean with *SEM*. tACS, transcranial alternating current stimulation; tDCS, transcranial direct current stimulation; BOLD, blood oxygen level dependent.

During the online fMRI n-back task performance, participants who received tACS exhibited larger brain activity compared to those who received sham in the three contrasts of interest. At the lowest and intermediate WM loads, more brain activity was found bilaterally in frontal regions, within the PCU, and in numerous widespread cortical (i.e., occipital) and subcortical (i.e., parahippocampal) regions. For the highest WM load, greater brain activity was only found bilaterally within the frontal pole (Figure 5A). Furthermore, the tACS group exhibited larger brain activity in frontal areas, the PCU, and within the lIPL, when compared to the tDCS group, in the intermediate and highest WM loads (Figure 5B). No significant differences were found when comparing tDCS with sham at this tES time-point.

#### Differential Post-stimulation Effects

**FIGURE 6:**
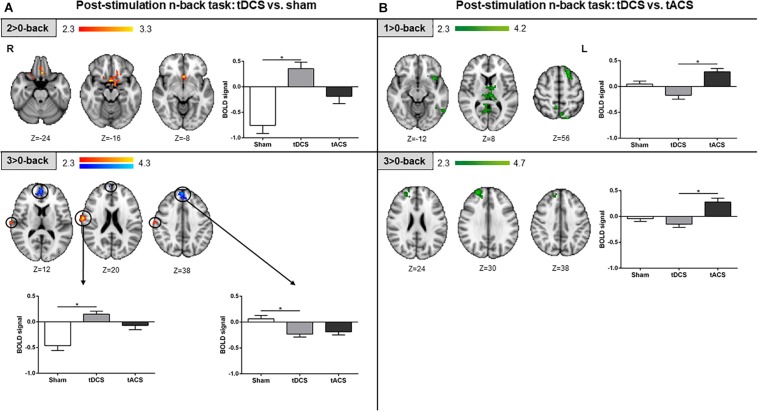
Post-stimulation fMRI n-back task results. **(A)** tDCS vs. sham results are shown in red-yellow for higher activations and blue-light blue for lower activations. **(B)** tDCS vs. tACS results are shown in green. Left and up: statistically significant fMRI activity maps on the standard MNI for each contrast of interest and group comparison. Right and below: plots of mean BOLD signal values at the fMRI clusters considering sham, tDCS and tACS where significant differences between groups were found. Data are presented as mean with *SEM*. tDCS, transcranial direct current stimulation; tACS, transcranial alternating current stimulation; BOLD, blood oxygen level dependent.

During the post-stimulation fMRI n-back task performance, differences in brain activity between tDCS and sham were detected. Specifically, in the intermediate WM load, the subcallosal cortex was found to be significantly more active in the tDCS than in the sham group. For the highest WM load, more fMRI signal was detected in a minor area of the right post-central gyrus in the tDCS group as compared to sham. Additionally, for the same WM load condition, the tDCS group showed significant lower brain activity within medial frontal structures when compared to sham (Figure 6A). When comparing tDCS vs. tACS, less brain activity was found in tDCS at the lowest WM load in distinct cortical (i.e., frontal and parietal) and subcortical (i.e., thalamic) areas. At the highest WM load, subjects who received tDCS showed lower activity as compared to tACS in right frontal areas only (Figure 6B). No significant differences were found when comparing tACS with sham at this tES time-point.

### Associations Between WM-Related Neural Activity and Performance

**FIGURE 7:**
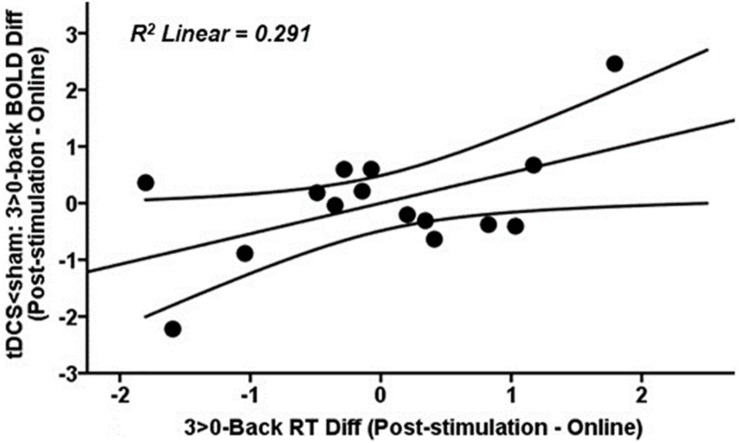
Scatter plot showing the relationship at the highest WM load between tDCS-induced changes in BOLD signal post-stimulation when compared to online within the fMRI cluster where tDCS showed less activity than sham after stimulation (Figure 6A, blue-light blue fMRI cluster) and RT post-stimulation as compared to online. Data are presented with z scores. tDCS, transcranial direct current stimulation; BOLD, blood oxygen level dependent; Diff, difference; RT, reaction time.

At the highest WM load, those tDCS subjects with a greater reduction in brain activity post-stimulation compared to online within the fMRI cluster where tDCS showed less activity than sham post-stimulation (Figure 6A, blue-light blue fMRI cluster) showed faster reaction time (RT) post-stimulation as compared to online performance (*r* = 0.540, *p* = 0.038; Figure 7). Faster responses were not associated with lower accuracy (i.e., d′; *p* > 0.05). No significant associations were detected between brain activity and performance in the tACS group (all *p* values > 0.05).

### tES-Related Individual Variability

**FIGURE 8:**
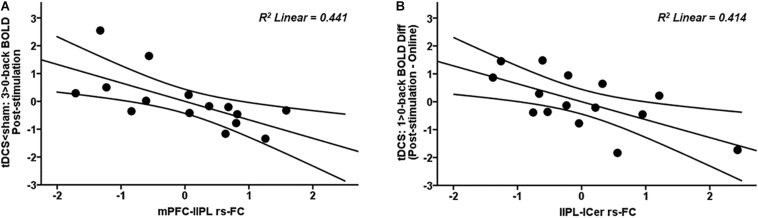
Scatter plots showing the relationships in the tDCS group between **(A)** mPFC-lIPL rs-FC and post-stimulation 3 > 0-back BOLD signal (in the tDCS < sham fMRI cluster) and **(B)** lIPL-lCer rs-FC and 1 > 0-back difference in BOLD signal (in the post-stimulation – online fMRI cluster). Data are presented with z scores. tDCS, transcranial direct current stimulation; BOLD, blood oxygen level dependent; Diff, difference; mPFC, medial prefrontal cortex; lIPL, left inferior parietal lobule; rs-FC, resting-state functional connectivity; lCer, left cerebellum.

Significant associations between rs-FC and brain activity were detected in the tDCS group. More precisely, those subjects of the tDCS group who exhibited higher mPFC-lIPL connectivity within the DMN also showed less BOLD signal at the highest WM load in the mPFC post-stimulation (Figure 6A; blue-light blue fMRI cluster; *r* = −0.664, *p* = 0.007; Figure 8A). Similarly, those subjects in the tDCS group displaying greater lIPL-left cerebellum connectivity within the DMN also showed a higher reduction in brain activity within the PCU at the lowest WM load post-stimulation as compared to online (Figure 4A; red-yellow fMRI cluster; *r* = −0.643, *p* = 0.010; Figure 8B). No significant relationships were observed between rs-FC and brain activity in the tACS group (all *p* values > 0.05).

### tES-Related Adverse Events

An interaction between experimental groups was found as regards tingling (*H* = 6.982, *p* = 0.030). Pairwise *post hoc* analyses revealed more tingling estimates in the tACS group as compared to sham (*p* = 0.025). Another interaction was found regarding phosphenes occurrence (χ*^2^* = 11.360, *p* = 0.003). Pairwise comparisons revealed a higher phosphenes occurrence in the tACS group in comparison to the tDCS group (*p* = 0.002, Fisher’s exact test; see Supplementary Table S1).

## Discussion

Our study employed fMRI connectivity and activity analyses to investigate, in an exploratory manner, the effects of two commonly used tES protocols over the WMN and its major anticorrelated circuit, the DMN. Albeit the usage of the same neurophysiological readout (i.e., fMRI) is a critical condition to be able to directly compare the effects of distinct stimulation modalities, this approach has seldom been used. To the best of our knowledge, there have only been two studies that compared the effects of tDCS and tACS on WM performance (Hoy et al., 2015; Röhner et al., 2018). However, these studies did not explore the distinct impact of those protocols on fMRI network dynamics. Our main results showed that using our tES montage: (1) prefrontal tDCS is capable of increasing rs-FC, mainly within the DMN, while prefrontal theta tACS appears to disrupt rs-fMRI systems. (2) Comparing both fMRI WM tasks, tDCS seem to exert its neural effects through a reduction on neural activity after stimulation, whilst tACS increase neural activity during stimulation, occurring both modulations mainly within DMN areas. (3) In the online fMRI WM task, tACS induced distributed neural activity which was not accommodated within the WMN, but overlapping certain posterior DMN areas. (4) In the post-stimulation fMRI WM task, tDCS strengthened expected medial prefrontal DMN deactivations, which correlated with faster responses. (5) Lastly, we observed that tDCS showed certain consistency on their neural effects across rs- and tb-fMRI, which was not the case for tACS.

### Effects of tDCS and tACS on rs-FC

We observed higher connectivity within the DMN in the tDCS group. This is in line with a previous investigation reporting rs-FC increases in this system after prefrontal tDCS (Keeser et al., 2011). This connectivity increase has been proposed to reflect augmented resources and higher readiness to facilitate cognition (Keeser et al., 2011). On the contrary, tACS appeared to reduce DMN and ECN rs-FC. Although relevant data about the tACS effects on rs-fMRI dynamics have recently been reported using distinct tES montages (i.e., Cabral-Calderin et al., 2016; Vosskuhl et al., 2016; Bächinger et al., 2017; Weinrich et al., 2017), prefrontal theta tACS impact on resting-state connectivity remains largely uninvestigated. The observed reduction in rs-FC for the tACS group could be explained as a disruption of the endogenous theta rhythm of the stimulated area. The theta frequency band mediates long-range connections in the brain through phase synchrony and cross-frequency coupling (for a review see Lisman and Jensen, 2013). Therefore, it is plausible that tACS at 6 Hz introduced an exogenous rhythm that disrupted phase synchrony on the DMN and ECN and thus decreased rs-FC.

### Effects of tDCS and tACS on WM-Related Neural Activity

#### Differential Effects Across tES Time-Points

In the present investigation, tDCS effects were primarily driven by reductions in neural activity after stimulation (Figure 4A). This is in line with the notion that some of the tDCS effects might take place after the cessation of stimulation rather than during stimulation, as observed in the motor cortex (Santarnecchi et al., 2014). On the contrary, tACS effects appeared to be particularly driven by changes during stimulation (Figure 4B). This is coherent with the outcome obtained using a similar protocol to ours, where a significant tACS effect on WM accuracy was only observed online, but not post-stimulation (Meiron and Lavidor, 2014). At the neuroanatomical level, the effects of both tES protocols on fMRI brain activity were mainly observed within relevant DMN areas, such as the mPFC and the PCU, the main hubs of this network (Buckner et al., 2008).

#### Differential Online Effects

In line with the abovementioned results, when exploring the neural modulations for each tES time-point, tACS effects were revealed to be different as compared to sham only in the online WM task (Figure 5A). Furthermore, neural activity changes were found in a cognitive load-dependent manner, were tACS engaged distributed neural resources in low load conditions, while at the highest demanding load, activity increases were only evident bifrontally. Notwithstanding, while the spatial distribution of the neuroimaging results did not completely overlap with the WM-dependent neural activity pattern, it plainly did meet some areas belonging to the DMN, such as the PCU. These tACS effects over the DMN were even more evident compared to tDCS (Figure 5B), where activity modulations in other DMN core areas also emerged, such as the lIPL. Altogether, prefrontal theta tACS during stimulation seemed to disrupt the characteristic WMN-DMN shift during externally oriented tasks (i.e., via a poorer DMN suppression, particularly within its posterior nodes). However, since no major significant behavioral differences were found between groups, this brain perturbation-like process (Paus, 2005) could have triggered compensatory overactivations in widespread regions regardless the WM-related areas, probably to allow a suitable response to the cognitive load required, and thus maintain cognition at an adequate level (i.e., such as the other experimental groups; see Supplementary Figure S1).

#### Differential Post-stimulation Effects

On the post-stimulation task, when compared to sham, we only detected brain activity changes in the tDCS group (Figure 6A). The most noteworthy outcome was a tDCS-induced activity reduction in a typically deactivated area corresponding to the anterior DMN node, namely the mPFC, in the highest WM load. It is worth noting that activity decreases are proportional to task difficulty (McKiernan et al., 2003). However, tDCS appeared to strengthen this characteristic medial prefrontal deactivation pattern. More interestingly, greater decreases in brain activity in this area were correlated with shorter response times (Figure 7). These results might be in line with the notion that tDCS increases neural efficiency on brain dynamics (Holland et al., 2011; Meinzer et al., 2012). In this case, neural efficiency may be operated via the inhibition of areas that need to be suppressed instead of an activity decrease in hyperactivated areas, as seen in normal and pathological aging (Meinzer et al., 2013, 2015). Thus, tDCS might be reinforcing the activation/deactivation WMN-DMN shift during the accomplishment of cognitive tasks, which is then associated with better performance.

### Cognitive Performance

Despite the brain-behavioral associations previously mentioned (see section “Differential Post-stimulation Effects”), we were not able to detect any cognitive effects between tES time-points and experimental groups. In this sense, although some studies with the same tDCS montage have reported significant improvements on WM performance both online and post-stimulation (Fregni et al., 2005; Ohn et al., 2008), more recent investigations have revealed that a single session of prefrontal tDCS does not (or at best, modestly) induce cognitive improvements in WM performance in healthy subjects (Brunoni and Vanderhasselt, 2014; Nilsson et al., 2015; Mancuso et al., 2016; Hill et al., 2017; Medina and Cason, 2017; Imburgio and Orr, 2018). This may be due to ceiling effects in healthy populations (Hsu et al., 2015). Regarding the effects of our tACS montage on cognitive performance, certain improvements in fluid intelligence have been previously observed (Pahor and Jaušovec, 2014), but not on WM (Jaušovec and Jaušovec, 2014). However, using slightly different tACS montages, either targeting prefrontal regions (Meiron and Lavidor, 2014; Alekseichuk et al., 2016) or the fronto-parietal circuit (Polanía et al., 2012; Violante et al., 2017), WM improvements have been reported. Yet, data in these regards is likewise inconsistent to date (Pahor and Jaušovec, 2018).

### tES-Related Individual Variability

Notable inter- and intra-individual variability has been seen in response to distinct non-invasive brain stimulation protocols (i.e., Hamada et al., 2013; López-Alonso et al., 2014; Martin-Trias et al., 2018). More specifically, López-Alonso et al. (2014) observed that only 45% of subjects responded as expected when the motor cortex was targeted with anodal tDCS. However, whether individual responses to different stimulation techniques are consistent across distinct fMRI modalities has not yet been explored. Our study is the first to investigate this issue. Our results indicate that in the tDCS group, those subjects displaying higher rs-FC in specific couplings also exhibited greater tb-fMRI modulations (Figure 8). Therefore, subjects that received tDCS seem to display consistency on their neural effects across different fMRI modalities, which seems not to be the case for tACS.

### Limitations

This investigation presents a number of limitations. First, although comparable to previous studies employing similar designs, the sample size was relatively modest. Second, an fMRI scan and a WM assessment at baseline (or using a cross-over design) could have permitted a more powerful analysis of the neural and cognitive effects of stimulation. Notwithstanding, to avoid practice effects, which previous WM studies have suffered (i.e., Röhner et al., 2018), a between-subjects design was selected. Further, in our study, methodological issues related to electrodes size or location might have underlie the absence of cognitive effects (for tDCS, see Imburgio and Orr, 2018; for tACS, see Mehta et al., 2015). Moreover, it is also worth noting that using individualized theta frequencies could have boosted cognitive performance in our experimental setting, although it does not guarantee any behavioral improvement by itself (Jaušovec and Jaušovec, 2014). Altogether, present data might provide a novel proof of concept of NIBS-fMRI effects for future research in the field. However, it is relevant to consider that our results should be interpreted in a cautious manner due to the stated limitations, which makes them exploratory and warranting further confirmation in larger studies.

## Conclusion

In sum, in the present investigation we have shown that prefrontal tDCS and tACS appear to display different effects on rs- and tb-fMRI neural dynamics. However, both particularly would affect DMN functioning at rest as well as during WM performance. In fact, the DMN is known to be highly susceptible to alterations by means of transcranial stimulation (i.e., Antonenko et al., 2018). This is crucial given that this network supports basic cognitive processes (Buckner et al., 2008). In addition, it is negatively affected both by advancing age (Andrews-Hanna et al., 2007; Chen et al., 2016; Schultz et al., 2017; Staffaroni et al., 2018) and in distinct neuropsychiatric conditions (Beucke et al., 2014; Hu et al., 2017; Wise et al., 2017; Yan et al., 2019). Indeed, its capability to being deactivated during externally oriented tasks is a process strongly affected both in aging (Miller et al., 2008) and disease (Fryer et al., 2013). In this vein, present results, along with those of other investigations allowing a better understanding of how the distinct DMN mechanisms -and its relationships with other systems- can be extrinsically modulated might provide valuable knowledge for future applications in both basic research and clinical care.

## Data Availability Statement

The data that support the findings of this study are available from the corresponding author upon reasonable request.

## Ethics Statement

The studies involving human participants were reviewed and approved by University of Barcelona’s Bioethics Commission (IRB 00003099). The patients/participants provided their written informed consent to participate in this study.

## Author Contributions

KA-P, LV-A, and DB-F designed the research and wrote the manuscript. KA-P and LV-A analyzed the data. KA-P, LV-A, RP-A, NB, M-FK, AP-L, MN, and DB-F edited the manuscript.

## Conflict of Interest

AP-L serves on the scientific advisory boards for Starlab Neuroscience, Neuroelectrics, Axilum Robotics, Constant Therapy, NovaVision, Cognito, Magstim, Nexstim, and Neosync, and is listed as an inventor on several issued and pending patents on the real-time integration of transcranial magnetic stimulation with electroencephalography and magnetic resonance imaging. MN serves on the scientific advisory board for Neuroelectrics. The remaining authors declare that the research was conducted in the absence of any commercial or financial relationships that could be construed as a potential conflict of interest.

## Footnotes

1 http://fsl.fmrib.ox.ac.uk/fsl/fslwiki/

2 https://afni.nimh.nih.gov/

## References

[B1] Abellaneda-Pérez K. Vaqué-Alcázar L. Vidal-Piñeiro D. Jannati A. Solana E. Bargalló N. (2019). Age-related differences in default-mode network connectivity in response to intermittent theta-burst stimulation and its relationships with maintained cognition and brain integrity in healthy aging. *Neuroimage* 188 794–806. 10.1016/j.neuroimage.2018.11.036 30472372 PMC6401253

[B2] Alekseichuk I. Turi Z. Amador de Lara G. Antal A. Paulus W. (2016). Spatial working memory in humans depends on theta and high gamma synchronization in the prefrontal cortex. *Curr. Biol.* 26 1513–1521. 10.1016/j.cub.2016.04.035 27238283

[B3] Ali M. M. Sellers K. K. Frohlich F. (2013). Transcranial alternating current stimulation modulates large-scale cortical network activity by network resonance. *J. Neurosci.* 33 11262–11275. 10.1523/JNEUROSCI.5867-12.2013 23825429 PMC6618612

[B4] Altman D. G. Bland J. M. (1999). How to randomise. *BMJ* 319 703–704. 10.1136/bmj.319.7211.703 10480833 PMC1116549

[B5] Anderson N. D. Craik F. I. (2017). 50 years of cognitive aging theory. *J. Gerontol. B Psychol. Sci. Soc. Sci.* 72 1–6. 10.1093/geronb/gbw108 27974471 PMC5156496

[B6] Andrews-Hanna J. R. Snyder A. Z. Vincent J. L. Lustig C. Head D. Raichle M. E. (2007). Disruption of large-scale brain systems in advanced aging. *Neuron* 56 924–935. 10.1016/j.neuron.2007.10.038 18054866 PMC2709284

[B7] Antal A. Herrmann C. S. (2016). Transcranial alternating current stimulation and transcranial random noise stimulation: possible mechanisms. *Neural Plast.* 2016:3616807. 10.1155/2016/3616807 27242932 PMC4868897

[B8] Antal A. Paulus W. (2013). Transcranial alternating current stimulation (tACS). *Front. Hum. Neurosci.* 7:317. 10.3389/fnhum.2013.00317 23825454 PMC3695369

[B9] Antonenko D. Külzow N. Sousa A. Prehn K. Grittner U. Flöel A. (2018). Neuronal and behavioral effects of multi-day brain stimulation and memory training. *Neurobiol. Aging* 61 245–254. 10.1016/j.neurobiolaging.2017.09.017 29050849

[B10] Bächinger M. Zerbi V. Moisa M. Polania R. Liu Q. Mantini D. (2017). Concurrent tACS-fMRI reveals causal influence of power synchronized neural activity on resting state fMRI connectivity. *J. Neurosci.* 37 4766–4777. 10.1523/JNEUROSCI.1756-16.2017 28385876 PMC6596494

[B11] Baddeley A. (1992). Working memory. *Science* 255 556–559. 10.1126/science.1736359 1736359

[B12] Baddeley A. (2010). Working memory. *Curr. Biol.* 20 136–140. 10.1016/j.cub.2009.12.014 20178752

[B13] Barbey A. K. Koenigs M. Grafman J. (2013). Dorsolateral prefrontal contributions to human working memory. *Cortex* 49 1195–1205. 10.1016/j.cortex.2012.05.022 22789779 PMC3495093

[B14] Beckmann C. F. DeLuca M. Devlin J. T. Smith S. M. (2005). Investigations into resting-state connectivity using independent component analysis. *Philos. Trans. R. Soc. Lond. B Biol. Sci.* 360 1001–1013. 10.1098/rstb.2005.1634 16087444 PMC1854918

[B15] Beucke J. C. Sepulcre J. Eldaief M. C. Sebold M. Kathmann N. Kaufmann C. (2014). Default mode network subsystem alterations in obsessive-compulsive disorder. *Br. J. Psychiatry* 205 376–382. 10.1192/bjp.bp.113.137380 25257066

[B16] Bikson M. Grossman P. Thomas C. Zannou A. L. Jiang J. Adnan T. (2016). Safety of transcranial direct current stimulation: evidence based update 2016. *Brain Stimul.* 9 641–661. 10.1016/j.brs.2016.06.004 27372845 PMC5007190

[B17] Brauer H. Kadish N. E. Pedersen A. Siniatchkin M. Moliadze V. (2018). No modulatory effects when stimulating the right inferior frontal gyrus with continuous 6 Hz tACS and tRNS on response inhibition: a behavioral study. *Neural Plast.* 2018:3156796. 10.1155/2018/3156796 30425735 PMC6218719

[B18] Brunoni A. R. Amadera J. Berbel B. Volz M. S. Rizzerio B. G. Fregni F. (2011). A systematic review on reporting and assessment of adverse effects associated with transcranial direct current stimulation. *Int. J. Neuropsychopharmacol.* 14 1133–1145. 10.1017/S1461145710001690 21320389

[B19] Brunoni A. R. Vanderhasselt M. A. (2014). Working memory improvement with non-invasive brain stimulation of the dorsolateral prefrontal cortex: a systematic review and meta-analysis. *Brain Cogn.* 86 1–9. 10.1016/j.bandc.2014.01.008 24514153

[B20] Buckner R. L. Andrews-Hanna J. R. Schacter D. L. (2008). The brain’s default network: anatomy, function, and relevance to disease. *Ann. N. Y. Acad. Sci.* 1124 1–38. 10.1196/annals.1440.011 18400922

[B21] Cabral-Calderin Y. Williams K. A. Opitz A. Dechent P. Wilke M. (2016). Transcranial alternating current stimulation modulates spontaneous low frequency fluctuations as measured with fMRI. *Neuroimage* 141 88–107. 10.1016/j.neuroimage.2016.07.005 27393419

[B22] Chen G. Shu H. Chen G. Ward B. D. Antuono P. G. Zhang Z. (2016). Staging alzheimer’s disease risk by sequencing brain function and structure, cerebrospinal fluid, and cognition biomarkers. *J. Alzheimers Dis.* 54 983–993. 10.3233/JAD-160537 27567874 PMC5055443

[B23] Curtis C. E. D’Esposito M. (2003). Persistent activity in the prefrontal cortex during working memory. *Trends Cogn. Sci.* 7 415–423. 10.1016/S1364-6613(03)00197-9 12963473

[B24] Dedoncker J. Brunoni A. R. Baeken C. Vanderhasselt M. A. (2016). A systematic review and meta-analysis of the effects of transcranial direct current stimulation (tDCS) over the dorsolateral prefrontal cortex in healthy and neuropsychiatric samples: influence of stimulation parameters. *Brain Stimul.* 9 501–517. 10.1016/j.brs.2016.04.006 27160468

[B25] Eriksson J. Vogel E. K. Lansner A. Bergström F. Nyberg L. (2015). Neurocognitive architecture of working memory. *Neuron* 88 33–46. 10.1016/j.neuron.2015.09.020 26447571 PMC4605545

[B26] Fertonani A. Ferrari C. Miniussi C. (2015). What do you feel if I apply transcranial electric stimulation? Safety, sensations and secondary induced effects. *Clin. Neurophysiol.* 126 2181–2188. 10.1016/j.clinph.2015.03.015 25922128

[B27] Fox M. D. Snyder A. Z. Vincent J. L. Corbetta M. Van Essen D. C. Raichle M. E. (2005). The human brain is intrinsically organized into dynamic, anticorrelated functional networks. *Proc. Natl. Acad. Sci. U.S.A.* 102 9673–9678. 10.1073/pnas.0504136102 15976020 PMC1157105

[B28] Fregni F. Boggio P. S. Nitsche M. Bermpohl F. Antal A. Feredoes E. (2005). Anodal transcranial direct current stimulation of prefrontal cortex enhances working memory. *Exp. Brain Res.* 166 23–30. 10.1007/s00221-005-2334-6 15999258

[B29] Fryer S. L. Woods S. W. Kiehl K. A. Calhoun V. D. Pearlson G. D. Roach B. J. (2013). Deficient suppression of default mode regions during working memory in individuals with early psychosis and at clinical high-risk for psychosis. *Front. Psychiatry* 4:92. 10.3389/fpsyt.2013.00092 24032017 PMC3768116

[B30] Hamada M. Murase N. Hasan A. Balaratnam M. Rothwell J. C. (2013). The role of interneuron networks in driving human motor cortical plasticity. *Cereb. Cortex* 23 1593–1605. 10.1093/cercor/bhs147 22661405

[B31] Hanslmayr S. Axmacher N. Inman C. S. (2019). Modulating human memory via entrainment of brain oscillations. *Trends Neurosci.* 42 485–499. 10.1016/j.tins.2019.04.004 31178076

[B32] Herrmann C. S. Rach S. Neuling T. Strüber D. (2013). Transcranial alternating current stimulation: a review of the underlying mechanisms and modulation of cognitive processes. *Front. Hum. Neurosci.* 7:279. 10.3389/fnhum.2013.00279 23785325 PMC3682121

[B33] Hill A. T. Rogasch N. C. Fitzgerald P. B. Hoy K. E. (2017). Effects of prefrontal bipolar and high-definition transcranial direct current stimulation on cortical reactivity and working memory in healthy adults. *Neuroimage* 152 142–157. 10.1016/j.neuroimage.2017.03.001 28274831

[B34] Holland R. Leff A. P. Josephs O. Galea J. M. Desikan M. Price C. J. (2011). Speech facilitation by left inferior frontal cortex stimulation. *Curr. Biol.* 21 1403–1407. 10.1016/j.cub.2011.07.021 21820308 PMC3315006

[B35] Howard M. W. Rizzuto D. S. Caplan J. B. Madsen J. R. Lisman J. Aschenbrenner-Scheibe R. (2003). Gamma oscillations correlate with working memory load in humans. *Cereb. Cortex* 13 1369–1374. 10.1093/cercor/bhg084 14615302

[B36] Hoy K. E. Bailey N. Arnold S. Windsor K. John J. Daskalakis Z. J. (2015). The effect of γ-tACS on working memory performance in healthy controls. *Brain Cogn.* 101 51–56. 10.1016/j.bandc.2015.11.002 26580743

[B37] Hsu W. Ku Y. Zanto T. P. Gazzaley A. (2015). Effects of non-invasive brain stimulation on cognitive function in healthy aging and Alzheimer’s disease: a systematic review and meta-analysis. *Neurobiol. Aging* 36 2348–2359. 10.1016/j.neurobiolaging.2015.04.016 26022770 PMC4496249

[B38] Hu M. L. Zong X. F. Mann J. J. Zheng J. J. Liao Y. H. Li Z. C. (2017). A review of the functional and anatomical default mode network in schizophrenia. *Neurosci. Bull.* 33 73–84. 10.1007/s12264-016-0090-1 27995564 PMC5567552

[B39] Imburgio M. J. Orr J. M. (2018). Effects of prefrontal tDCS on executive function: methodological considerations revealed by meta-analysis. *Neuropsychologia* 117 156–166. 10.1016/j.neuropsychologia.2018.04.022 29727626

[B40] Jaušovec N. Jaušovec K. (2014). Increasing working memory capacity with theta transcranial alternating current stimulation (tACS). *Biol. Psychol.* 96 42–47. 10.1016/j.biopsycho.2013.11.006 24291565

[B41] Jenkinson M. Beckmann C. F. Behrens T. E. J. Woolrich M. W. Smith S. M. (2012). FSL. *Neuroimage* 62 782–790. 10.1016/j.neuroimage.2011.09.015 21979382

[B42] Jensen O. Colgin L. L. (2007). Cross-frequency coupling between neuronal oscillations. *Trends Cogn. Sci.* 11 267–269. 10.1016/j.tics.2007.05.003 17548233

[B43] Johnson M. K. McMahon R. P. Robinson B. M. Harvey A. N. Hahn B. Leonard C. J. (2013). The relationship between working memory capacity and broad measures of cognitive ability in healthy adults and people with schizophrenia. *Neuropsychology* 27 220–229. 10.1037/a0032060 23527650 PMC3746349

[B44] Kang M. Ragan B. G. Park J. H. (2008). Issues in outcomes research: an overview of randomization techniques for clinical trials. *J. Athl. Train.* 43 215–221. 10.4085/1062-6050-43.2.215 18345348 PMC2267325

[B45] Kasten F. H. Dowsett J. Herrmann C. S. (2016). Sustained aftereffect of α-tACS lasts up to 70 min after stimulation. *Front. Hum. Neurosci.* 10:245. 10.3389/fnhum.2016.00245 27252642 PMC4879138

[B46] Keeser D. Meindl T. Bor J. Palm U. Pogarell O. Mulert C. (2011). Prefrontal transcranial direct current stimulation changes connectivity of resting-state networks during fMRI. *J. Neurosci.* 31 15284–15293. 10.1523/JNEUROSCI.0542-11.2011 22031874 PMC6703525

[B47] Lang S. Gan L. S. Alrazi T. Monchi O. (2019). Theta band high definition transcranial alternating current stimulation, but not transcranial direct current stimulation, improves associative memory performance. *Sci. Rep.* 9:8562. 10.1038/s41598-019-44680-8 31189985 PMC6561937

[B48] Lee J. Park S. (2005). Working memory impairments in schizophrenia: a meta-analysis. *J. Abnorm. Psychol.* 114 599–611. 10.1037/0021-843X.114.4.599 16351383

[B49] Liebetanz D. Nitsche M. A. Tergau F. Paulus W. (2002). Pharmacological approach to the mechanisms of transcranial DC-stimulation-induced after-effects of human motor cortex excitability. *Brain* 125 2238–2247. 10.1093/brain/awf238 12244081

[B50] Lisman J. E. Jensen O. (2013). The theta-gamma neural code. *Neuron* 77 1002–1016. 10.1016/j.neuron.2013.03.007 23522038 PMC3648857

[B51] López-Alonso V. Cheeran B. Río-Rodríguez D. Fernández-Del-Olmo M. (2014). Inter- individual variability in response to non-invasive brain stimulation paradigms. *Brain Stimul.* 7 372–380. 10.1016/j.brs.2014.02.004 24630849

[B52] Mancuso L. E. Ilieva I. P. Hamilton R. H. Farah M. J. (2016). Does transcranial direct current stimulation improve healthy working memory? a meta-analytic review. *J. Cogn. Neurosci.* 28 1063–1089. 10.1162/jocn_a_00956 27054400

[B53] Martin-Trias P. Lanteaume L. Solana E. Cassé-Perrot C. Fernández-Cabello S. Babiloni C. (2018). Adaptability and reproducibility of a memory disruption rTMS protocol in the PharmaCog IMI European project. *Sci. Rep.* 8:9371. 10.1038/s41598-018-27502-1 29921865 PMC6008461

[B54] Matsumoto H. Ugawa Y. (2017). Adverse events of tDCS and tACS: a review. *Clin. Neurophysiol. Pract.* 2 19–25. 10.1016/j.cnp.2016.12.003 30214966 PMC6123849

[B55] McKiernan K. A. Kaufman J. N. Kucera-Thompson J. Binder J. R. (2003). A parametric manipulation of factors affecting task-induced deactivation in functional neuroimaging. *J. Cogn. Neurosci.* 15 394–408. 10.1162/089892903321593117 12729491

[B56] Medina J. Cason S. (2017). No evidential value in samples of transcranial direct current stimulation (tDCS) studies of cognition and working memory in healthy populations. *Cortex* 94 131–141. 10.1016/j.cortex.2017.06.021 28759803

[B57] Mehta A. R. Pogosyan A. Brown P. Brittain J. S. (2015). Montage matters: the influence of transcranial alternating current stimulation on human physiological tremor. *Brain Stimul.* 8 260–268. 10.1016/j.brs.2014.11.003 25499037 PMC4319690

[B58] Meinzer M. Antonenko D. Lindenberg R. Hetzer S. Ulm L. Avirame K. (2012). Electrical brain stimulation improves cognitive performance by modulating functional connectivity and task-specific activation. *J. Neurosci.* 32 1859–1866. 10.1523/JNEUROSCI.4812-11.2012 22302824 PMC6703352

[B59] Meinzer M. Lindenberg R. Antonenko D. Flaisch T. Flöel A. (2013). Anodal transcranial direct current stimulation temporarily reverses age-associated cognitive decline and functional brain activity changes. *J. Neurosci.* 33 12470–12478. 10.1523/JNEUROSCI.5743-12.2013 23884951 PMC6618670

[B60] Meinzer M. Lindenberg R. Phan M. T. Ulm L. Volk C. Flöel A. (2015). Transcranial direct current stimulation in mild cognitive impairment: behavioral effects and neural mechanisms. *Alzheimers Dement.* 11 1032–1040. 10.1016/j.jalz.2014.07.159 25449530

[B61] Meiron O. Lavidor M. (2014). Prefrontal oscillatory stimulation modulates access to cognitive control references in retrospective metacognitive commentary. *Clin. Neurophysiol.* 125 77–82. 10.1016/j.clinph.2013.06.013 23831184

[B62] Miller S. L. Celone K. DePeau K. Diamond E. Dickerson B. C. Rentz D. (2008). Age-related memory impairment associated with loss of parietal deactivation but preservd hippocampal activation. *Proc. Natl. Acad. Sci. U.S.A.* 105 2181–2186. 10.1073/pnas.0706818105 18238903 PMC2538895

[B63] Moisa M. Polania R. Grueschow M. Ruff C. C. (2016). Brain network mechanisms underlying motor enhancement by transcranial entrainment of gamma oscillations. *J. Neurosci.* 36 12053–12065. 10.1523/JNEUROSCI.2044-16.2016 27881788 PMC6604912

[B64] Monte-Silva K. Kuo M. F. Hessenthaler S. Fresnoza S. Liebetanz D. Paulus W. (2013). Induction of late LTP-like plasticity in the human motor cortex by repeated non-invasive brain stimulation. *Brain Stimul.* 6 424–432. 10.1016/j.brs.2012.04.011 22695026

[B65] Nakao T. Nakagawa A. Nakatani E. Nabeyama M. Sanematsu H. Yoshiura T. (2009). Working memory dysfunction in obsessive-compulsive disorder: a neuropsychological and functional MRI study. *J. Psychiatr. Res.* 43 784–791. 10.1016/j.jpsychires.2008.10.013 19081580

[B66] Nee D. E. Brown J. W. Askren M. K. Berman M. G. Demiralp E. Krawitz A. (2013). A Meta-analysis of executive components of working memory. *Cereb. Cortex* 23 264–282. 10.1093/cercor/bhs007 22314046 PMC3584956

[B67] Nilsson J. Lebedev A. V. Lovden M. (2015). No significant effect of prefrontal tDCS on working memory performance in older adults. *Front. Aging Neurosci.* 7:230. 10.3389/fnagi.2015.00230 26696882 PMC4677281

[B68] Nitsche M. A. Cohen L. G. Wassermann E. M. Priori A. Lang N. Antal A. (2008). Transcranial direct current stimulation: State of the art 2008. *Brain Stimul.* 1 206–223. 10.1016/j.brs.2008.06.004 20633386

[B69] Nitsche M. A. Fricke K. Henschke U. Schlitterlau A. Liebetanz D. Lang N. (2003). Pharmacological modulation of cortical excitability shifts induced by transcranial direct current stimulation in humans. *J Physiol.* 553 293–301. 10.1113/jphysiol.2003.049916 12949224 PMC2343495

[B70] Nitsche M. A. Paulus W. (2000). Excitability changes induced in the human motor cortex by weak transcranial direct current stimulation. *J. Physiol.* 527 633–639. 10.1111/j.1469-7793.2000.t01-1-00633.x 10990547 PMC2270099

[B71] Nitsche M. A. Paulus W. (2001). Sustained excitability elevations induced by transcranial DC motor cortex stimulation in humans. *Neurology* 57 1899–1901. 10.1212/WNL.57.10.1899 11723286

[B72] Ohn S. H. Park C. I. Yoo W. K. Ko M. H. Choi K. P. Kim G. M. (2008). Time-dependent effect of transcranial direct current stimulation on the enhancement of working memory. *Neuroreport* 19 43–47. 10.1097/WNR.0b013e3282f2adfd 18281890

[B73] Owen A. M. McMillan K. M. Laird A. R. Bullmore E. (2005). N-back working memory paradigm: a meta-analysis of normative functional neuroimaging studies. *Hum. Brain Mapp.* 25 46–59. 10.1002/hbm.20131 15846822 PMC6871745

[B74] Pahor A. Jaušovec N. (2014). The effects of theta transcranial alternating current stimulation (tACS) on fluid intelligence. *Int. J. Psychophysiol.* 93 322–331. 10.1016/j.ijpsycho.2014.06.015 24998643

[B75] Pahor A. Jaušovec N. (2018). The effects of theta and gamma tACS on working memory and electrophysiology. *Front. Hum. Neurosci.* 11:651. 10.3389/fnhum.2017.00651 29375347 PMC5767723

[B76] Park D. C. Lautenschlager G. Hedden T. Davidson N. S. Smith A. D. Smith P. K. (2002). Models of visuospatial and verbal memory across the adult life span. *Psychol. Aging* 17 299–320. 10.1037/0882-7974.17.2.299 12061414

[B77] Park D. C. Reuter-Lorenz P. (2009). The adaptive brain: aging and neurocognitive scaffolding. *Annu. Rev. Psychol.* 60 173–196. 10.1146/annurev.psych.59.103006.093656 19035823 PMC3359129

[B78] Paus T. (2005). Inferring causality in brain images: a perturbation approach. *Philos. Trans. R. Soc. Lond. B Biol. Sci.* 360 1109–1114. 10.1098/rstb.2005.1652 16087451 PMC1854935

[B79] Peña-Gómez C. Sala-Lonch R. Junqué C. Clemente I. Vidal D. Bargalló N. (2012). Modulation of large-scale brain networks by transcranial direct current stimulation evidenced by resting-state functional MRI. *Brain Stimul.* 5 252–263. 10.1016/j.brs.2011.08.006 21962981 PMC3589751

[B80] Pfefferbaum A. Chanraud S. Pitel A. L. Muller-Oehring E. Shankaranarayanan A. Alsop D. C. (2011). Cerebral blood flow in posterior cortical nodes of the default mode network decreases with task engagement but remains higher than in most brain regions. *Cereb. Cortex* 21 233–244. 10.1093/cercor/bhq090 20484322 PMC3000573

[B81] Polanía R. Nitsche M. A. Korman C. Batsikadze G. Paulus W. (2012). The importance of timing in segregated theta phase-coupling for cognitive performance. *Curr. Biol.* 22 1314–1318. 10.1016/j.cub.2012.05.021 22683259

[B82] Polanía R. Nitsche M. A. Ruff C. C. (2018). Studying and modifying brain function with non-invasive brain stimulation. *Nat. Neurosci.* 21 174–187. 10.1038/s41593-017-0054-4 29311747

[B83] Purpura D. P. McMurtry J. G. (1965). Intracellular activities and evoked potential changes during polarization of motor cortex. *J. Neurophysiol.* 28 166–185. 10.1152/jn.1965.28.1.166 14244793

[B84] Raichle M. E. (2015). The brain’s default mode network. *Annu. Rev. Neurosci.* 38 433–447. 10.1146/annurev-neuro-071013-014030 25938726

[B85] Reato D. Rahman A. Bikson M. Parra L. C. (2013). Effects of weak transcranial alternating current stimulation on brain activity—a review of known mechanisms from animal studies. *Front. Hum. Neurosci.* 7:687. 10.3389/fnhum.2013.00687 24167483 PMC3805939

[B86] Röhner F. Breitling C. Rufener K. S. Heinze H. J. Hinrichs H. Krauel K. (2018). Modulation of working memory using transcranial electrical stimulation: a direct comparison between TACS and TDCS. *Front. Neurosci.* 12:761. 10.3389/fnins.2018.00761 30405341 PMC6206050

[B87] Roux F. Uhlhaas P. J. (2014). Working memory and neural oscillations: alpha-gamma versus theta-gamma codes for distinct WM information? *Trends Cogn. Sci.* 18 16–25. 10.1016/j.tics.2013.10.010 24268290

[B88] Sala-Llonch R. Peña-Gómez C. Arenaza-Urquijo E. M. Vidal-Piñeiro D. Bargalló N. Junqué C. (2012). Brain connectivity during resting state and subsequent working memory task predicts behavioural performance. *Cortex* 48 1187–1196. 10.1016/j.cortex.2011.07.006 21872853

[B89] Santarnecchi E. Feurra M. Barneschi F. Acampa M. Bianco G. Cioncoloni D. (2014). Time course of corticospinal excitability and autonomic function interplay during and following monopolar tDCS. *Front. Psychiatry* 5:86. 10.3389/fpsyt.2014.00086 25101009 PMC4104833

[B90] Sarnthein J. Petsche H. Rappelsberger P. Shaw G. L. von Stein A. (1998). Synchronization between prefrontal and posterior association cortex during human working memory. *Proc. Natl. Acad. Sci. U.S.A.* 95 7092–7096. 10.1073/pnas.95.12.7092 9618544 PMC22750

[B91] Sauseng P. Klimesch W. Schabus M. Doppelmayr M. (2005). Fronto-parietal EEG coherence in theta and upper alpha reflect central executive functions of working memory. *Int. J. Psychophysiol.* 57 97–103. 10.1016/j.ijpsycho.2005.03.018 15967528

[B92] Schultz A. P. Chhatwal J. P. Hedden T. Mormino E. C. Hanseeuw B. J. Sepulcre J. (2017). Phases of hyperconnectivity and hypoconnectivity in the default mode and salience networks track with amyloid and tau in clinically normal individuals. *J. Neurosci.* 37 4323–4331. 10.1523/JNEUROSCI.3263-16.2017 28314821 PMC5413178

[B93] Smith S. M. Fox P. T. Miller K. L. Glahn D. C. Fox P. M. Mackay C. E. (2009). Correspondence of the brain’s functional architecture during activation and rest. *Proc. Natl. Acad. Sci. U.S.A.* 106 13040–13045. 10.1073/pnas.0905267106 19620724 PMC2722273

[B94] Smith S. M. Jenkinson M. Woolrich M. W. Beckmann C. F. Behrens T. E. J. Johansen-Berg H. (2004). Advances in functional and structural MR image analysis and implementation as FSL. *Neuroimage* 23(Suppl. 1), S208–S219. 10.1016/j.neuroimage.2004.07.051 15501092

[B95] Staffaroni A. M. Brown J. A. Casaletto K. B. Elahi F. M. Deng J. Neuhaus J. (2018). The longitudinal trajectory of default mode network connectivity in healthy older adults varies as a function of age and is associated with changes in episodic memory and processing speed. *J. Neurosci.* 38 2809–2817. 10.1523/JNEUROSCI.3067-17.2018 29440553 PMC5852659

[B96] Tremblay S. Lepage J. F. Latulipe-Loiselle A. Fregni F. Pascual-Leone A. Théoret H. (2014). The uncertain outcome of prefrontal tDCS. *Brain Stimul.* 7 773–783. 10.1016/j.brs.2014.10.003 25456566 PMC4342747

[B97] Unsworth N. Fukuda K. Awh E. Vogel E. K. (2014). Working memory and fluid intelligence: capacity, attention control, and secondary memory retrieval. *Cogn. Psychol.* 71 1–26. 10.1016/j.cogpsych.2014.01.003 24531497 PMC4484859

[B98] Violante I. R. Li L. M. Carmichael D. W. Lorenz R. Leech R. Hampshire A. (2017). Externally induced frontoparietal synchronization modulates network dynamics and enhances working memory performance. *eLife* 6:e22001. 10.7554/eLife.22001 28288700 PMC5349849

[B99] Vossen A. Gross J. Thut G. (2015). Alpha power increase after transcranial alternating current stimulation at alpha frequency (a-tACS) reflects plastic changes rather than entrainment. *Brain Stimul.* 8 499–508. 10.1016/j.brs.2014.12.004 25648377 PMC4464304

[B100] Vosskuhl J. Huster R. J. Herrmann C. S. (2016). BOLD signal effects of transcranial alternating current stimulation (tACS) in the alpha range: a concurrent tACS–fMRI study. *Neuroimage* 140 118–125. 10.1016/j.neuroimage.2015.10.003 26458516

[B101] Weinrich C. A. Brittain J. S. Nowak M. Salimi-Khorshidi R. Brown P. Stagg C. J. (2017). Modulation of long-range connectivity patterns via frequency-specific stimulation of human cortex. *Curr. Biol.* 27 3061.e3–3068.e3. 10.1016/j.cub.2017.08.075 28966091 PMC5640151

[B102] Wischnewski M. Engelhardt M. Salehinejad M. A. Schutter D. J. L. G. Kuo M. F. Nitsche M. A. (2019). NMDA receptor-mediated motor cortex plasticity after 20 Hz transcranial alternating current stimulation. *Cereb. Cortex* 29 2924–2931. 10.1093/cercor/bhy160 29992259

[B103] Wise T. Marwood L. Perkins A. M. Herane-Vives A. Joules R. Lythgoe D. J. (2017). Instability of default mode network connectivity in major depression: a two-sample confirmation study. *Transl. Psychiatry* 7:e1105. 10.1038/tp.2017.40 28440813 PMC5416685

[B104] Woods A. J. Antal A. Bikson M. Boggio P. S. Brunoni A. R. Celnik P. (2016). A technical guide to tDCS, and related non-invasive brain stimulation tools. *Clin. Neurophysiol.* 127 1031–1048. 10.1016/j.clinph.2015.11.012 26652115 PMC4747791

[B105] Woolrich M. W. Behrens T. E. Beckmann C. F. Jenkinson M. Smith S. M. (2004). Multilevel linear modelling for FMRI group analysis using Bayesian inference. *Neuroimage* 21 1732–1747. 10.1016/j.neuroimage.2003.12.023 15050594

[B106] Woolrich M. W. Ripley B. D. Brady M. Smith S. M. (2001). Temporal autocorrelation in univariate linear modeling of FMRI data. *Neuroimage* 14 1370–1386. 10.1006/nimg.2001.0931 11707093

[B107] Yan C. G. Chen X. Li L. Castellanos F. X. Bai T. J. Bo Q. J. (2019). Reduced default mode network functional connectivity in patients with recurrent major depressive disorder. *Proc. Natl. Acad. Sci. U.S.A.* 116 9078–9083. 10.1073/pnas.1900390116 30979801 PMC6500168

[B108] Zaehle T. Sandmann P. Thorne J. D. Jäncke L. Herrmann C. S. (2011). Transcranial direct current stimulation of the prefrontal cortex modulates working memory performance: combined behavioural and electrophysiological evidence. *BMC Neurosci.* 12:2. 10.1186/1471-2202-12-2 21211016 PMC3024225

[B109] Zaghi S. Acar M. Hultgren B. Boggio P. S. Fregni F. (2010a). Noninvasive brain stimulation with low-intensity electrical currents: putative mechanisms of action for direct and alternating current stimulation. *Neuroscientist* 16 285–307. 10.1177/1073858409336227 20040569

[B110] Zaghi S. de Freitas Rezende L. de Oliveira L. M. El-Nazer R. Menning S. Tadini L. (2010b). Inhibition of motor cortex excitability with 15Hz transcranial alternating current stimulation (tACS). *Neurosci. Lett.* 479 211–214. 10.1016/j.neulet.2010.05.060 20553804

