# Supplementary material for: Differential tDCS and tACS Effects on Working Memory-Related Neural Activity and Resting-State Connectivity
Source: Front Neurosci. 2020 Jan 17;13:1440. doi: 10.3389/fnins.2019.01440 (PMC6978675; doi:10.3389/fnins.2019.01440)
Supplement: Supplementary file 1 [file Table_1.DOCX]

Supplementary Material

# Materials and methods

tES-related adverse events assessment

At the end of the experimental session, all the participants were asked if any tES-related adverse event was experienced while the stimulation device was transferring current (i.e., during the rs-fMRI and the first tb-fMRI acquisitions). The adverse events studied were headache, neck pain, pain in the scalp, tingling, itching, burning sensation, local erythema, drowsiness, difficulty in concentration, and sharp mood swings. All the adverse events were classified as absent (0), mild (1), moderate (2) or severe (3). Additionally, phosphenes occurrence was recorded in a dichotomous manner (absence or presence).

Transcranial electrical stimulation (tES) parameters

Following previous tES-fMRI studies and the manufacturer’s safety guidelines, both electrodes were located with an orientation of their connectors to the midline of the head (i.e., Holland et al., 2011). The stimulator was situated outside the MRI room, and stimulation current was fed via two stages of radio frequency filtration to prevent inference being gathered by the scanner. The electrodes were covered with Ten20 conductive paste to ensure a comfortable fitting between the electrodes and the scalp surface, thus optimizing conductivity and minimizing impedance.

N-back task

We used a block-designed task where each n-back condition lasted for 26 s, followed by inter-block fixation periods of 13 s. Before any n-back block, an instruction screen was shown to inform the subject about the upcoming block. Each stimulus (capital letter A-J) was presented in white in the center of a black screen during 500 ms, with an inter-stimulus interval of 1,500 ms. Subjects were instructed to press a button when the letter shown matched the one seen one (1-back), two (2-back) or three (3-back) stimuli before or when the letter ‘X’ appeared (0-back). The individual performance was recorded and scores were calculated using the d prime (d’) measure -which accounts for correct responses and false alarms-, computed as: Z(hit rate) - Z(false alarm rate), where function Z(p), p ∈ [0,1], is the inverse of the cumulative distribution function of the Gaussian distribution of the hits and false alarms rates. Mean RT and total number of hits in each condition were also recorded (Sala-Llonch et al., 2012).

Functional connectivity analyses

Rs-fMRI data preprocessing included removal of the first 5 volumes, motion correction, skull stripping, spatial smoothing [Full Width at Half Maximum (FWHM) = 7 mm], grand mean scaling and filtering with both high-pass and low-pass filters (0.1- and 0.01-Hz thresholds, respectively). The data were then regressed with six rigid-body realignment motion parameters, mean white matter and mean cerebrospinal fluid signal. No global signal regression was used. Registration to an MNI standard space was performed through a two-step linear transformation.

Moreover, as head movement may affect rs-fMRI data (Power et al., 2012; 2015; van Dijk et al., 2012), two standard measures to estimate in-scanner head motion were obtained in a similar way as described elsewhere (Power et al., 2012). Displacement relative to a single reference volume (absolute displacement) and relative to the precedent volume (relative displacement) were calculated for every subject. No significant differences were found between groups in any of the two measures (all *p* values > 0.05).

N-back fMRI data

The preprocessing of task-related fMRI scans included non-brain tissue removal, motion correction, spatial smoothing with a Gaussian kernel of 5 mm of FWHM, temporal high pass filter of 160 s application, and a linear registration to an MNI standard template carried out using FLIRT (Jenkinson & Smith, 2001; Jenkinson et al., 2002).

With respect to in-scanner head motion, any warning for excessive movement was reported after the first level analysis for each subject. Further, there were no significant interactions between tES time-points and groups in absolute or relative displacement (all *p* values > 0.05).

Statistical analyses

Data distribution in non-imaging analyses was tested for normality with the Shapiro-Wilk test (*p* > 0.05; Razali & Wah, 2011; Shapiro & Wilk, 1965).

For group comparisons in non-imaging analyses containing non-parametric data, the Kruskal-Wallis *H* test (for *k* independent samples) or Mann-Whitney *U* test (for 2 independent samples) or Wilcoxon test (for 2 related samples) were used. Pairwise post-hoc analyses for the Kruskal-Wallis *H* test were subjected to Dunn-Bonferroni correction. For categorical data, the chi-squared (χ^2^) test (for *k* independent samples) or Fisher's exact test (for 2 independent samples) or McNemar’s test (for 2 related samples) were used. When data introduced in correlational analyses were not normally distributed, a Spearman’s correlation was performed.

In all statistical analyses (i.e., imaging and non-imaging), results were only reported when both the interaction and the pairwise post-hoc analyses were statistically significant.

**Results**

Demographics and n-back task performance


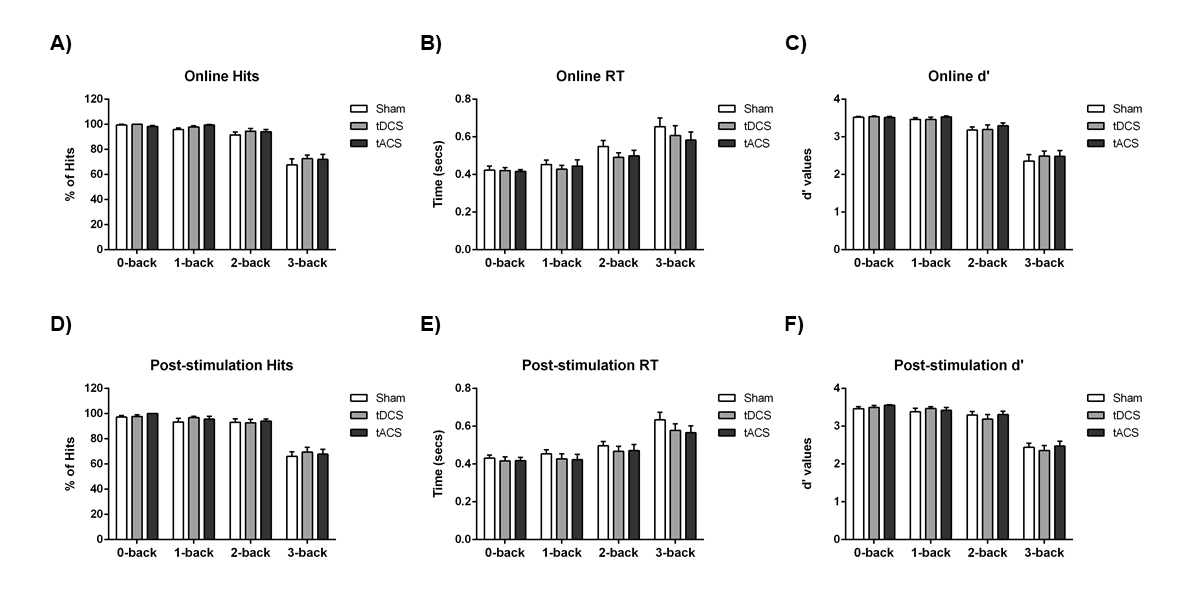


**Fig. S1.** N-back performance in the online and post-stimulation fMRI tasks for hits (A and D, respectively), RT (B and E, respectively) and d’ (C and F, respectively). Abbreviations: tDCS, transcranial direct current stimulation; tACS, transcranial alternating current stimulation; RT, reaction time; d’, d prime.

Effects of tDCS and tACS on WM-related neural activity

**
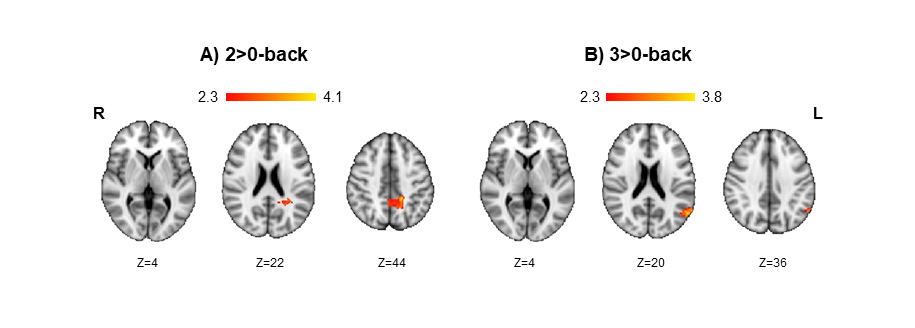
**

**Fig. S2.** Results from the interaction between tES time-points and experimental groups in A) 2>0-back and B) 3>0-back contrasts. Statistically significant fMRI activity maps are shown in red-yellow on the standard MNI.


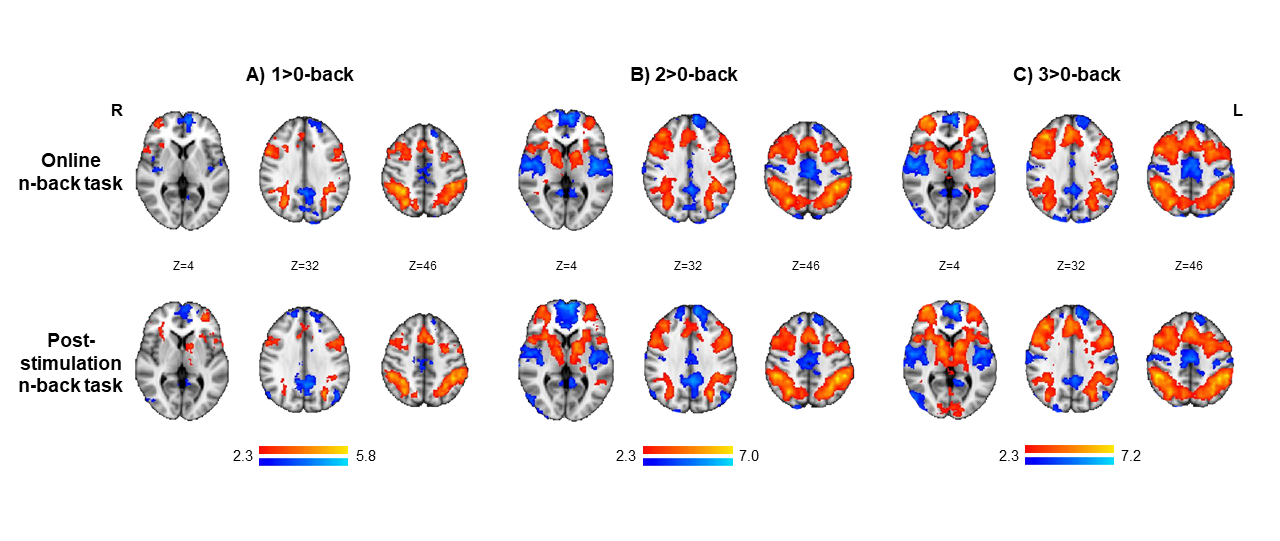


**Fig. S3.** Group-mean activity maps obtained from each contrast: A) 1>0-back; B) 2>0-back and C) 3>0-back at the online (top) and post-stimulation (down) tES time-points in the sham group. The WM patterns for the contrasts of interest are shown in red-yellow and task-deactivations are shown in blue-light blue on the standard MNI.

tES-related adverse events

|  | **Total (N = 44)** | **Sham (N = 15)** | **tDCS (N = 15)** | **tACS (N = 14)** |
| --- | --- | --- | --- | --- |
| **Headache** | 0.27 ± 0.76 | 0.47 ± 1.13 | 0.27 ± 0.59 | 0.07 ± 0.27 |
| **Neck pain** | 0.34 ± 0.78 | 0.53 ± 0.99 | 0.33 ± 0.82 | 0.14 ± 0.36 |
| **Pain in the scalp** | 0.14 ± 0.46 | 0.20 ± 0.56 | 0.07 ± 0.26 | 0.14 ± 0.53 |
| **Tingling** | 0.68 ± 0.71 | 0.33 ± 0.49 | 0.67 ± 0.62 | 1.07 ± 0.83 |
| **Itching** | 0.36 ± 0.61 | 0.33 ± 0.72 | 0.47 ± 0.64 | 0.29 ± 0.47 |
| **Burning sensation** | 0.68 ± 0.91 | 0.53 ± 0.74 | 0.80 ± 1.08 | 0.71 ± 0.91 |
| **Local erythema** | 0.30 ± 0.55 | 0.13 ± 0.35 | 0.53 ± 0.64 | 0.21 ± 0.58 |
| **Drowsiness** | 0.73 ± 1.09 | 1.20 ± 1.37 | 0.47 ± 0.99 | 0.50 ± 0.65 |
| **Difficulty in concentration** | 0.55 ± 0.82 | 0.73 ± 1.03 | 0.73 ± 0.80 | 0.14 ± 0.36 |
| **Sharp mood swings** | 0.14 ± 0.55 | 0.20 ± 0.77 | 0.20 ± 0.56 | 0.00 ± 0.00 |
| **Phosphenes occurrence**  **(absence/presence)** | 30/14 | 11/4 | 14/1 | 5/9 |

**Table S1.** tES-related adverse events estimates. Data are presented as mean ± *SD* for the whole sample and considering the three experimental groups. Data represents the mean intensity of adverse events from a 0-3 scale. Abbreviations: tDCS, transcranial direct current stimulation; tACS, transcranial alternating current stimulation.

**References**

Holland, R., Leff, A. P., Josephs, O., Galea, J. M., Desikan, M., Price, C. J., et al. (2011). Speech facilitation by left inferior frontal cortex stimulation. *Current Biology*, *21*(16), 1403–1407. http://doi.org/10.1016/j.cub.2011.07.021

Jenkinson, M. & Smith, S. (2001). A Global optimisation method for robust affine registration of brain images. *Medical Image Analysis*, *5*(2), 143–156. http://doi.org/10.1016/S1361-8415(01)00036-6

Jenkinson, M., Bannister, P., Brady, M. & Smith, S. (2002). Improved optimisation for the robust and accurate linear registration and motion correction of brain images. *NeuroImage, 17*(2), 825–841. https://doi.org/10.1006/nimg.2002.1132

Power, J. D., Barnes, K. A., Snyder, A. Z., Schlaggar, B. L., & Petersen, S. E. (2012). Spurious but systematic correlations in functional connectivity MRI networks arise from subject motion. *NeuroImage*, *59*(3), 2142–2154. http://doi.org/10.1016/j.neuroimage.2011.10.018

Power, J. D., Schlaggar, B. L., & Petersen, S. E. (2015). Recent progress and outstanding issued in motion correction resting state fMRI. *NeuroImage*, *105*, 536–551. http://doi.org/10.1016/j.neuroimage.2014.10.044

Razali, N. M., & Wah, Y. B. (2011). Power comparisons of Shapiro-Wilk , Kolmogorov-Smirnov, Lilliefors and Anderson-Darling tests. *Journal of Statistical Modeling and Analytics*, *2*(1), 21–33.

Sala-Llonch, R., Peña-Gómez, C., Arenaza-Urquijo, E. M., Vidal-Piñeiro, D., Bargalló, N., Junqué, C. & Bartrés-Faz, D. (2012). Brain connectivity during resting state and subsequent working memory task predicts behavioural performance. *Cortex*, *48*(9), 1187–1196. http://doi.org/10.1016/j.cortex.2011.07.006

Shapiro, S. S. & Wilk, M. B. (1965). An Analysis of Variance Test for Normality (Complete Samples). *Biometrika*, *52*(3/4), 591–611. https://doi.org/10.2307/2333709

van Dijk, K. R. A., Sabuncu, M. R. & Buckner, R. L. (2012). The influence of head motion on intrinsic functional connectivity MRI. *NeuroImage*, *59*(1), 431–438. http://doi.org/10.1016/j.neuroimage.2011.07.044
